# Electrodeposited reduced graphene oxide-PEDOT:PSS/Nafion hybrid interface for the simultaneous determination of dopamine and serotonin

**DOI:** 10.1038/s41598-023-47693-6

**Published:** 2023-11-20

**Authors:** Seung Hyeon Ko, Seung Wook Kim, Soo Hyun Lee, Yi Jae Lee

**Affiliations:** 1 Brain Science Institute, Korea Institute of Science and Technology, Seoul, 02792 South Korea; 2 Department of Chemical and Biological Engineering, Korea University, Seoul, 02841 South Korea; 3 Division of Bio-Medical Science & Technology, KIST School, University of Science & Technology (UST), Seoul, 02792 South Korea

**Keywords:** Diagnosis, Biomedical materials, Biomedical engineering

## Abstract

The electrochemically deposited reduced graphene oxide-PEDOT:PSS/Nafion (rGO-PP/NF) hybrid material has provided a favorable interface for the simultaneous detection of dopamine (DA) and serotonin (5-HT). The rGO-PP/NF onto the Au seed layer of the flexible substrate was simple, and it was followed by the sequential electrophoretic deposition of GO, reduction at the optimal pH buffer media, electropolymerization of EDOT:PSS, and Nafion coating. The strong electron-transport capacity between rGO-PEDOT:PSS and the negatively charged Nafion matrix might allow the highly sensitive, simultaneous, and selective detection of DA and 5-HT due to its high affinity for cations. In the results of the electrochemical response, well-separated oxidation peaks were observed in a mixture that contained various concentrations of DA and 5-HT. It showed the dynamic sensing of DA and 5-HT in the ranges of 0.5–75 μM and 0.05–50 μM, respectively, and the detection limits of 0.17 and 0.16 μM, respectively. In the mixture of DA and 5-HT, the sensor had a detection limit of 0.1 μM for 5-HT and DA, and its sensitivities of DA and 5-HT were 99.3 and 86 µA/µMcm^2^. Furthermore, it demonstrated high selectivity, reproducibility, stability, and a recovery property in the human serum spike test that was good enough for the practical use.

## Introduction

Neurotransmitters are important in various neural systems that have crucial roles between brain neurons, as well as maintaining neural functions. The dysregulation of neurotransmitters can cause several brain disorders. An imbalance of neurotransmitters has been associated with physiological and psychological diseases. Among all of the neurotransmitters, dopamine (DA) and serotonin (5-HT, 5-hydroxytryptamine) are monoamine neurotransmitters that are distributed extensively throughout the human body and brain. These neurotransmitters have a vital role in the regulation of numerous behavioral and physiological functions^1^. Deficiencies in the levels of these neurotransmitters can cause mood-related disorders, depression, migraine headaches, sexual disorders, and Parkinson’s disease. For these reasons, the monitoring of DA and 5-HT is of great significance in the diagnosis of various diseases.

Electrochemical techniques are known to be very sensitive detection tools, and they also allow simultaneous quantitative analysis. Many electrochemical sensors have been reported, including sensors related to 5-HT. However, the advancement in the electrochemical 5-HT determination and the simultaneous detection of 5-HT has been hindered by interfering signals of DA due to the similar redox potentials. In addition, the detection of 5-HT in physiological samples can be affected adversely by electroactive metabolites, which not only make the systems both insensitive and non-selective but also can make trouble in stability and reproducibility of the sensor^2,3^. However, an effective solution is to modify the electrode with conductive or catalytic materials to improve the sensitivity and selectivity of the target detection. Therefore, the introduction of nanomaterial-modified electrodes as a well-established strategy can provide a favorable interface to achieve highly selective, sensitive neurotransmitters detection.

Recently, carbon nanomaterials have been used to improve the electro-catalytic ability and selectivity of biosensors. Graphene oxide (GO) has attracted significant attention for various applications due to its high specific surface area and unique structure which increase the sensitivity of the sensor as catalytic support^4^ even though it still is lacking in selectivity.

Since DA and 5-HT detections rely on the electro-catalytic properties of the sensor, the electrode material with high conduction property and favorable electroactive interface can crucial role. Thus, nanomaterial-based composites could serve an important role in constructing a highly sensitive sensor. In our previous work, we demonstrated that a flexible sensor consisting of the GO/PEDOT:PSS composite could provide a feasible surface for the sensitive and selective detection of DA^5^. This hybrid composite provided larger surface area and it also facilitated the selective determination of small, active molecules (e.g. AA, DA, UA) due to its net-negative charge interface. GO has a unique ability to act as a versatile dispersant, and it is highly biocompatible and has good electrocatalytic properties^6^. However, the oxygen-containing groups on the GO sheets allow it to swell readily and be dispersed in water and some other solvents^7^. Thus, these functional groups of GO can cause poor stability in the analyses of real samples.

In this work, the optimal electrochemical reduction condition for constructing reduced graphene oxide (rGO) with a high electroactive interface property was based on an extension of the patterned GO surface that we reported previously. In general, chemically reduced graphene oxide has been used extensively as a reduction method that uses reducing agents, such as hydrazine^8^, hydroxylamine^9^, and hydroquinone^10^. The excessive use of reducing agent could contaminate the resulting product and even be harmful to human health and the environment^11^. Unlike the chemical reduction method, electrochemical reduction provides a facile, fast, scalable, economic, and environmentally benign pathway to the production of graphene and related materials^12^. After preparation of optimal rGO, the EDOT:PSS was electro-polymerized onto the surface of the rGO, which improved the electro-active surface area and provided enhanced pathways for movement of electrons by the oxidation of analytes. It has the important role of providing for the highly sensitive and selective detection of DA and 5-HT.

The sensor with the rGO-PEDOT:PSS/Nafion (rGO-PP/NF) electrode was characterized its electrochemical performance by electrochemical impedance spectroscopy (EIS), cyclic voltammetry (CV), and differential pulse voltammetry (DPV) for the sensing of DA and 5-HT. The surface morphology, chemical state, and elemental composition of the rGO-PP/NF were investigated using a scanning electron microscope (SEM), Fourier-transform infrared (FT-IR) spectroscopy, Raman spectra, and high-resolution X-ray photoelectron spectroscopy (XPS).

To the best of our knowledge, this is the first study that selectively deposited GO under low current onto a thin Au electrode for the flexible DA and 5-HT sensor, while most electrophoretically-deposited GO patterns have been fabricated under high potential or by the drop-coating of a prepared GO suspension. The electrophoretic deposition of GO under low current has some advantages, such as the easy and stable deposition on thin seed metal layers, thickness control, and uniformity. In addition, the pre-deposition of GO onto the surface of an electrode was reduced electrochemically in a non-hazardous aqueous buffer solution at room temperature. Since the decorated PEDOT:PSS by simple electropolymerization onto prepared rGO at the optimal pH and negatively charged Nafion coating^13^, the rGO-PEDOT:PSS/Nafion electrode exhibited significantly enhanced catalytic properties toward the electro-active detection of multi neurotransmitters.

## Results and discussion

### Characterization of the reduced GO electrodes

Although the GO can be reduced electrochemically over the wide pH range of 1.5–12.5, the optimal pH condition for the medium also is essential to ensure that the rGO surface has a good electro-catalytic property. The reduced GO film was prepared by cyclic voltammetry for the potential range from − 1.5 to 0 V in buffer solutions with various pH conditions.

**Figure 1:**
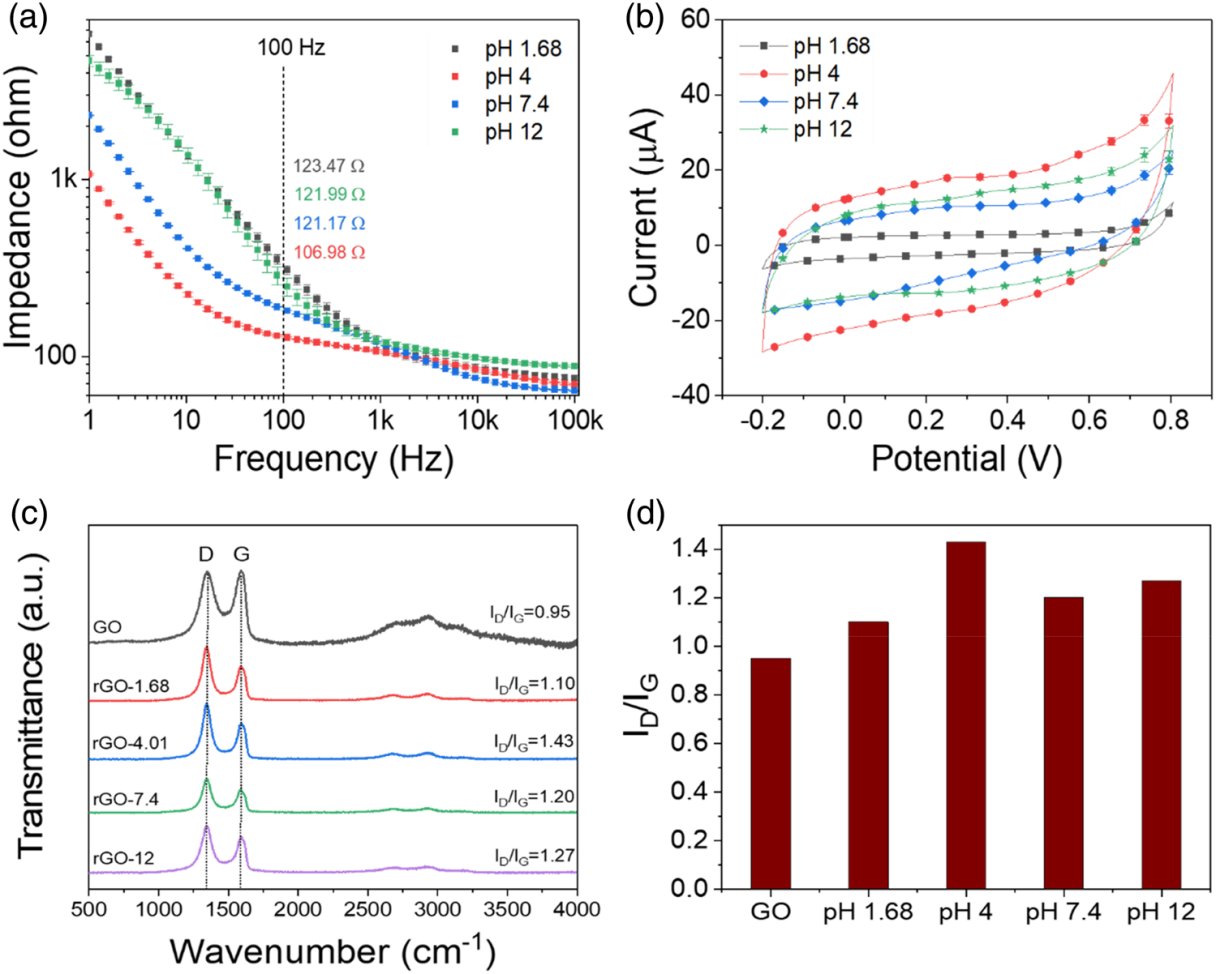
(**a**) EIS and (**b**) CV curve (scan rates of 100 mV/s) plot of the rGO electrode in 0.1 M PBS solution (pH 7.4) after the electrochemical reduction of the GO electrode at different pH solutions (1.68, 4, 7.4, and 12); (**c**) comparison of the Raman spectra for the fabricated rGO electrodes in solutions that have different pH values; (**d**) comparison of the I_D_/I_G_ ratio obtained from the Raman spectra of the GO and rGOs fabricated using solution that had different pH values.

Figure 1a and b show the interfacial impedance and CV curves of the rGO electrode fabricated with different pH conditions in a 0.1 M PBS solution. The electrochemical reduction was accomplished through the removal of the oxygen functional groups of GO, which recover the graphitic domains of the carbon bond. Thus, the concentration of H^+^ in the media has a strong impact on the performance of the rGO. As shown Fig. 1a and b, the interfacial impedances of the rGO fabricated at pH conditions of 1.68, 4, 7.4, and 12 were 123.47 ± 9.75, 106.98. ± 1.13, 121.17 ± 0.38, and 121.99 ± 5.14 Ω at the 100 Hz, and the charge storage capacity (CSC), i.e., the real activation area, was increased sharply at pH 4 and decreased at the other pH conditions (0.24, 1.33, 0.79, and 1.07 mC/cm^2^). The effect of the pH of the solution on the electrochemical reduction of GO has been reported by other researchers^9,14^. The electrochemical reduction at those ranges (pH < 2; pH > 10) showed a side-reaction, which can cause both competition with the reduction of H^+^ and the formation of hydrogen bubbles at the working electrode. Thus, the medium of the neural region provides a favorable condition for the reduction of GO without any interfering side-reactions. The pH of 4 was considered to be appropriate for electrochemical reduction.

According to the measured Raman spectroscopy to check the quantitative analysis of rGO in Fig. 1c, the characteristic D, G bands were represented by peaks at approximately 1345/cm and 1590/cm, respectively^15^. The D and G bands corresponded to the sp2 and sp3 carbon stretching modes. The intensity ratio of the D and G band (I_D_/I_G_) is a measure of the size of the sp2 ring in a domain of sp2 and sp3 carbon bonds, implying that the GO has been reduced successfully^16,17^. Figure 1d shows that the I_D_/I_G_ ratio of GO (ca. 0.95) increased significantly after the electrochemical reduction. The I_D_/I_G_ ratios of rGOs for pH values of 1.68, 4, 7.4, and 12 (i.e., 1.10, 1.43, 1.20, and 1.27, respectively) were higher than that of GO, indicating an increment in the number of smaller sp2 domains after electrochemical reduction. After reduction at the pH value of 4, the intensity ratio, i.e., (I_D_/I_G_) results from a lower defect density due to the rapid electrochemical reduction rate. These results also supports the electrochemical characterization properties in Fig. 1a and b, which show that the concentration of H^+^ has a strong affect on the electrochemical reduction, resulting in the improved performance of rGO. Thus, the pH 4 condition was selected as a buffer condition for rGO.

## Characterization of the rGO-PP/NF electrode

### Morphological analysis

**Figure 2:**
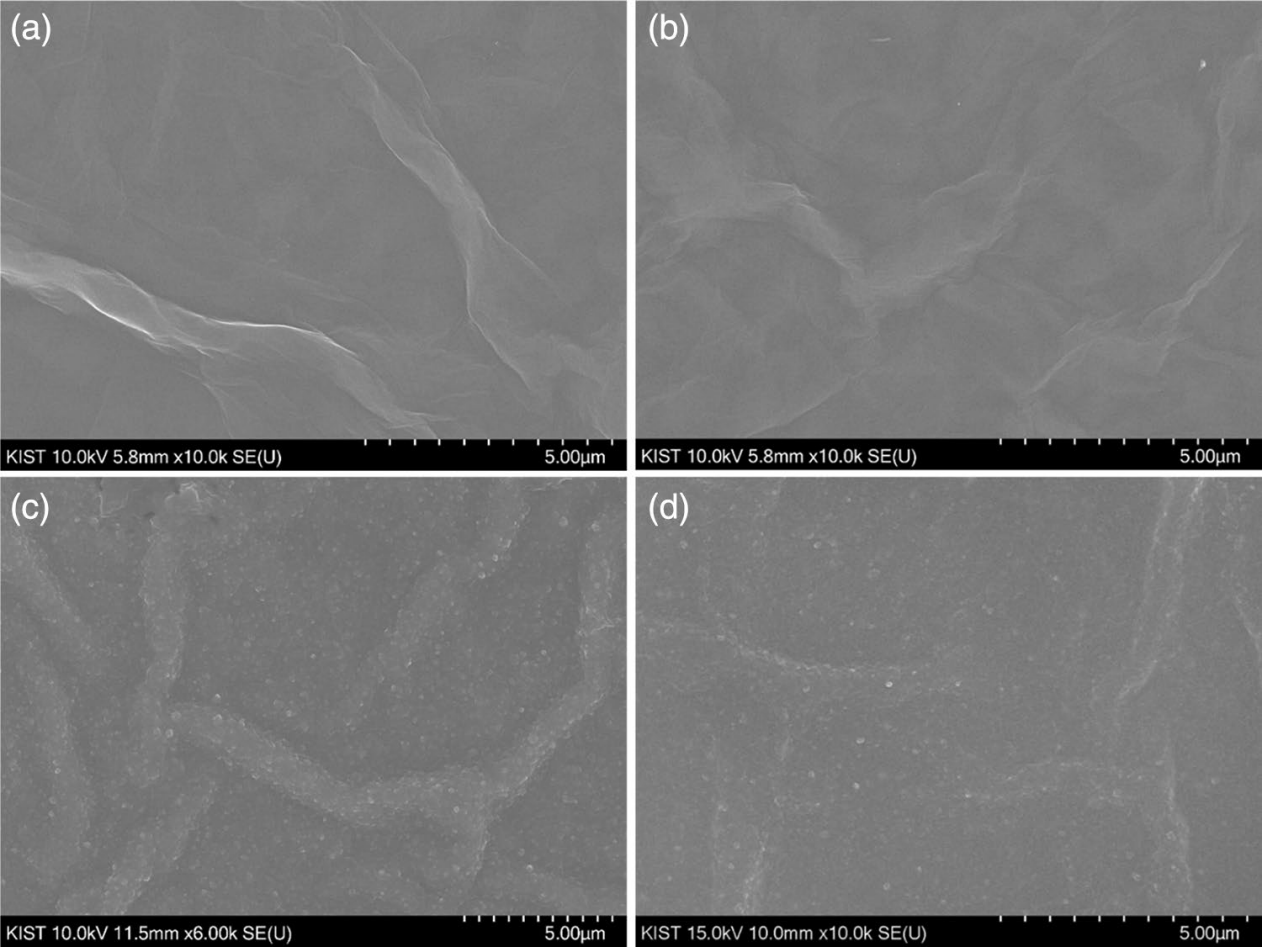
Scanning electron microscopy images of the fabricated entities: (**a**) GO, (**b**) rGO, (**c**) rGO-PEDOT:PSS, and (**d**) rGO-PEDOT:PSS/Nafion. All images display an 10,000 × magnification of the electrode surfaces. The scale bars represent 5 µm.

The surface morphologies of the fabricated GO, rGO, rGO-PP, and rGO-PP/NF were measured and compared with SEM in Fig. 2. The deposited GO (a) and rGO (b) layer onto the thin Au electrode exhibited random winkle structure. The surface of (c) rGO-PP and (d) rGO-PP/NF clearly showed different structure when compared to that of the structure of rGO, which seemed like covering by the well-distributed PEDOT:PSS along the ridges formed by the rGO. In addition, the surface of rGO-PP/NF seemed to look like rGO-PP, which indicates that the well distributed thin Nafion layer will not affect the structure of rGO-PP.

**Figure 3:**
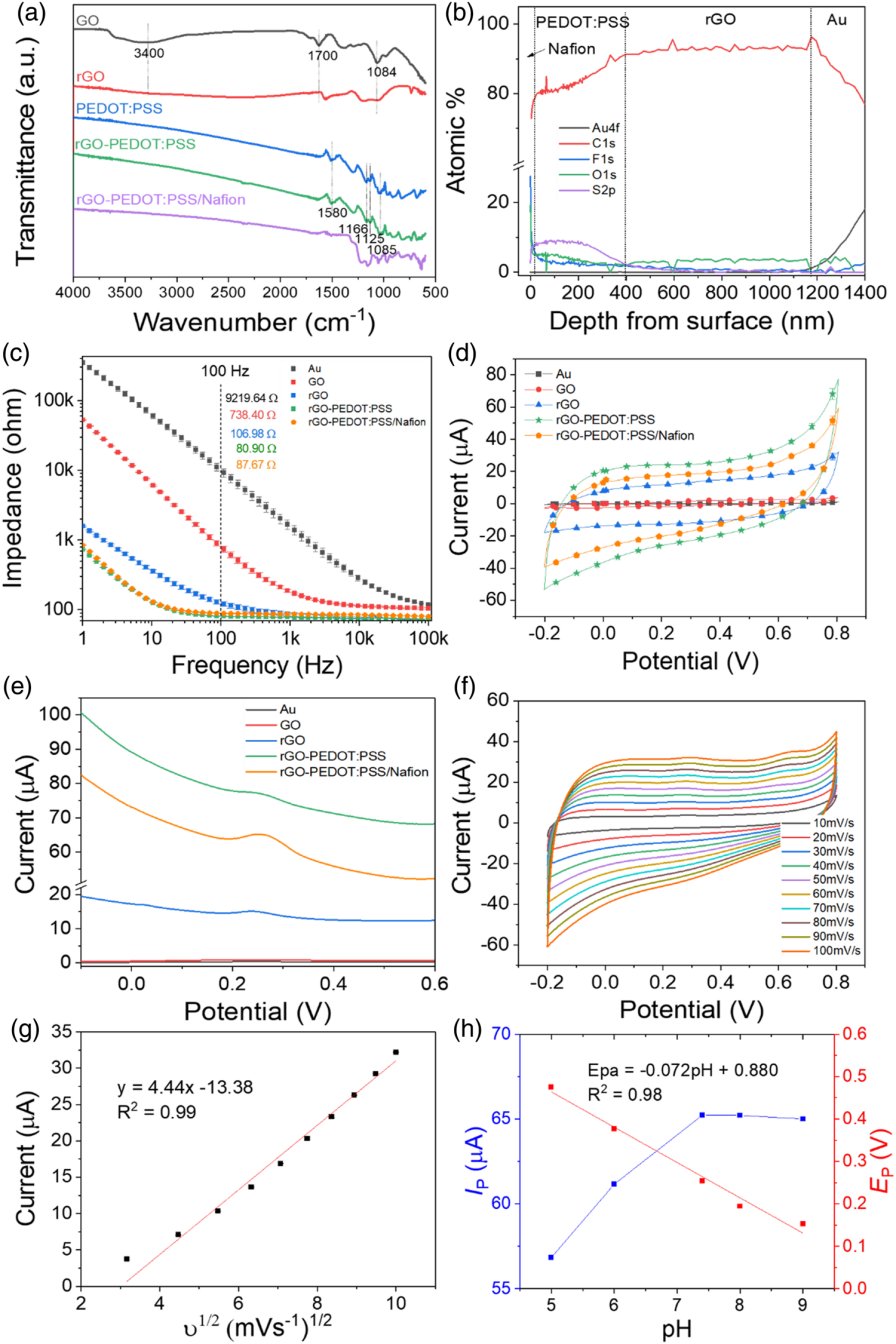
(**a**) FT-IR spectra of the GO, rGO, PEDOT:PSS, rGO-PEDOT:PSS, and rGO-PEDOT:PSS/Nafion electrode deposited on Au electrode; (**b**) XPS depth profile of rGO-PEDOT:PSS/Nafion electrode; (**c**) EIS and (**d**) CV curve (scan rate of 100 mV/s) plot of bare Au, GO, rGO, rGO-PEDOT:PSS, and rGO-PEDOT:PSS/Nafion electrode in 0.1 M PBS solution (pH 7.4); (**e**) DPV curve of bare Au, GO, rGO, rGO-PEDOT:PSS, and rGO-PEDOT:PSS/Nafion electrode containing 1 μM of 5-HT; (**f**) CV curve of rGO-PEDOT:PSS/Nafion electrode in 3 mM 5-HT to different scan rates from 10 to 100 mV/s; (**g**) A linear fit of CV oxidation peak currents of 5-HT concentration (n = 3); (**h**) effect of pH (pH ranging from 5.0 to 9.0) on DPV (scan rate of 25 mV/s) of the rGO-PEDOT:PSS/Nafion electrode in 0.1 M PBS with 1 μM serotonin. The relationship of pH vs. peak current (I_p_, blue line) and peak potential (E_p_, red line) for serotonin. Error bars represent standard deviation.

In Fig. 3a, the fabricated GO, rGO, rGO-PP, and rGO-PP/NF working electrode were investigated by FT-IR to verify the chemical modification. Figure 3a shows the removal of the oxygenated functional groups from rGO compared to GO. After electrochemical reduction, the oxygenated groups essentially were removed distinctly, as shown by the absence of transmittance of at 3400/cm, 1700/cm, and 1084/cm. This attributed to the O–H, C=O, and C–O stretching vibration, respectively. This indicates that most of the oxygen oxygenated groups in GO can be eliminated effectively by electrochemical reduction. The rGO-PP transmittance curve reflected on the formation of new bond by PEDOT:PSS that the vibrations at 1580/cm and 1508/cm (thiophene ring) and 1166/cm and 1125/cm (phenyl group) belong to the rGO-PP spectra^18,19^. This indicates that the PEDOT:PSS was well constructed on the surface of the rGO by electro-polymerization. Interestingly, the peak intensity of the rGO-PP/NF at 1500–1000/cm was increased significantly after coating of the Nafion layer, which was caused by symmetric C-F stretching at 1145/cm and asymmetric C-F stretching band at 1201/cm^20^.

In order to investigate the presence of layer of different materials in the rGO-PP/NF, a depth profiling study were performed for the level regions of Au4f, C1s, F1s, O1s, and S2p. Figure 3b shows the depth profile of XPS, and it can provide the approximate thickness of the each layer of the materials for the rGO-PP/NF due to the increment and decrement of each of the atomic concentrations and intensities that were measured. Therefore, the thickness of Nafion, PEDOT:PSS, and rGO were about 3, 400, and 800 nm, respectively. More detailed XPS analysis was added in Supplementary Fig. S1. The percentage of atomic carbon was decreased sharply at the surface between rGO and Au. This result was relatively well matched with the cross-sectional SEM image of the rGO-PP/NF in Supplementary Fig. S2.

### Electrochemical properties

In order to investigate the electrochemical performance of the fabricated working electrodes, the interfacial impedance and cyclic voltammogram of the Au, GO, rGO, rGO-PP and the rGO-PP/NF electrode in a 0.1 M PBS solution (pH 7.4) were measured and compared in the Fig. 3c and d. The measured interfacial impedance of the Au, GO, rGO, rGO-PP, and rGO-PP/NF electrodes at 100 Hz were 9219.64 ± 72.63, 738.40 ± 7.4, 360.11 ± 7.12, 80.90 ± 0.7, and 87.67 ± 0.57 Ω, respectively. The interfacial impedance of the rGO-PP electrode was enhanced extremely compared to the other electrodes due to good conductivity and the low resistance to charge transfer. PEDOT:PSS was an expanded current response that indicated an increase in the number of electrochemically active sites^21^. In the CV curve that was measured, the CSC value that means charges have accumulated was expanded gradually in the order of Au < GO < rGO < rGO-PP/NF < rGO-PP, the values of which were 0.15, 1.14, 13.3, 22.6, and 15.6 mC/cm^2^, respectively. After being electrochemically reduced and deposited, the rGO-PP was enhanced such that it had a higher capacitance value. With the addition of Nafion, the current response of the rGO-PP/NF was lowered more than that of rGO-PP, which might have been caused by hindering some active sites and the weakened catalytic activity^22^.

In the DPV response curve for the sensors that were fabricated with Au, GO, rGO, rGO-PP, and rGO-PP/NF to the 1 μM 5-HT of the Fig. 3e, it is difficult to discern of the peak responses for the sensor with bare Au and GO, while the oxidation peak responses for the sensor with rGO and rGO-PP were improved slightly. In the case of the sensor with rGO-PP/NF, the oxidation peak for 5-HT was observed clearly in comparison to those of the sensor with Au, rGO, and rGO-PP. This might have been caused by the attribution of the negatively charged Nafion for the strongly attracted cations, such as 5-HT.

### Effects of scan rate and pH

**Figure 4:**
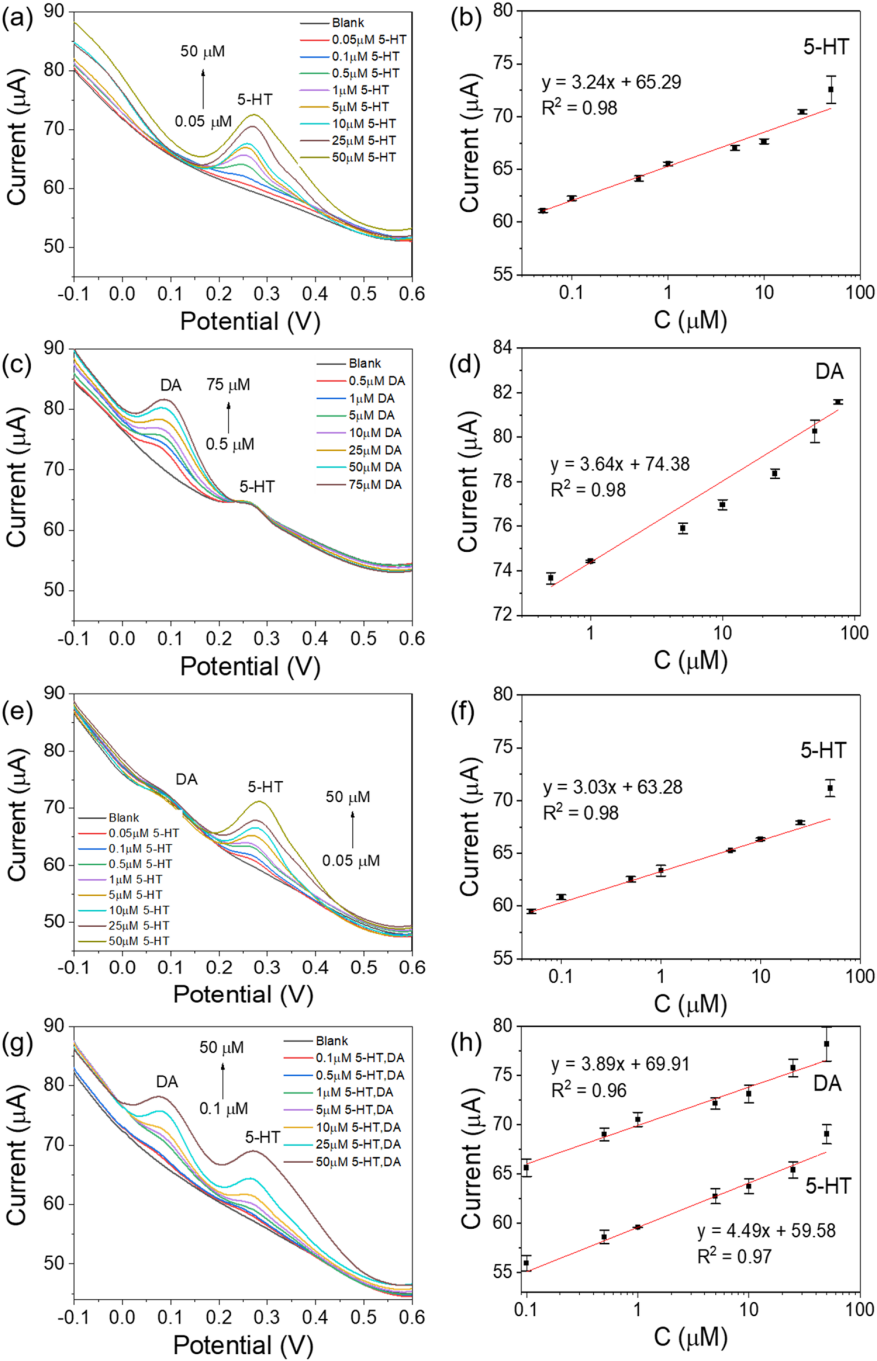
DPV responses of the rGO-PEDOT:PSS/Nafion electrode to the (**a**, **b**) different concentrations of 5-HT (0.05–50 μM) and its calibration plot, (**c**, **d**) 1 μM 5-HT with different concentration of DA (0.5–75 μM) and its calibration plot, (**e**, **f**) 1 μM DA with different concentration of 5-HT (0.05–50 μM), (**g**, **h**) simultaneously changed 5-HT and DA concentrations (0.1–50 μM) in 0.1 M PBS (pH 7.4).

The influence of scan rate was evaluated using the CV curve in Fig. 4f and g. The CV curves of rGO-PP/NF in Fig. 3f were recorded with scan rate from 10 to 100 mV/s in PBS (pH 7.4). Therefore, between the oxidation peak current and square root of the scan rate, good linear relationship (R^2^ = 0.98) were found in Fig. 3g. This result indicates that a typical diffusion-controlled electron transfer process occurred at rGO-PP/NF electrode.

The effect of pH on the oxidation of 5-HT at the sensor with rGO-PP/NF was examined with DPV in the pH range 5–9. Figure 3h shows the relationship between pH and the peak current during the oxidation of 5-HT. It shows that, when the pH is increased, the oxidation potential shifts negatively, and the highest peak current was obtained at a pH value of 7.4. So, maximum value of the current at pH 7.4 indicates the high electrochemical activity of 5-HT on rGO-PP/NF. The calculated regression equation is E_pa_ (V) = − 0.072 pH + 0.88 (R^2^ = 0.98). The slope was − 72 mV/pH, which was slightly different from the theoretical value of − 59 mV/pH. This confirms that two electrons and two protons were transferred in the oxidation process of 5-HT as indicated by the Nernst equation^23^. Hence, the pH value of 7.4 was chosen to be an appropriate value for further studies of 5-HT detection at the rGO-PP/NF.

### DPV responses of the sensor with rGO-PP/NF to the DA and 5-HT

As shown in Fig. 4a, the sensor with the rGO-PP/NF electrode was tested by using the DPV technique for the determination of 5-HT at various concentrations (0.05–50 μM) in 0.1 M PBS (pH 7.4). The oxidation peak current of the sensor with the rGO-PP/NF electrode was increased gradually by about 0.25 V as an increment of the 5-HT concentration from 0.05 to 50 μM. The linearity from the peak current (IP) of the sensor to the 5-HT concentration is shown in Fig. 4b. The linear regression equation for 5-HT can be expressed as IP (μA) = 3.24 C (μM) + 65.29 (R^2^ = 0.98). The sensitivity and the calculated limit of detection (LOD) of the sensor with rGO-PP/NF were 71.66 μA/μM cm^2^ and 0.17 μM, respectively.

For investing the selective detection of DA and 5-HT, one concentration was fixed and the other concentration was changed. The simultaneous detection of two analytes was conducted in the prepared mixture by simultaneously changing the concentration of the one specie. As shown in Fig. 4, the current responses for DA and 5-HT were exhibited at separate potential zones (0.09 V and 0.25 V) with a good linearity. The sensitivity for DA and 5-HT was 80.5 and 67.1 μA/μM cm^2^, and it showed an calculated LOD value of 0.17 for DA and 0.16 μM for 5-HT, respectively, in the dynamic ranges of 0.5–75 μM DA at fixed 1 μM 5-HT (Fig. 4c) and 0.05–50 μM 5-HT at fixed 1 μM DA (Fig. 4e), which are good enough for clinical use. In Fig. 4(g), the sensor with rGO-PP/NF also exhibited linear current response with increasing concentration of DA and 5-HT. In addition, the correlation linear response (Fig. 4d, f and h), only showed a small change, which indicated good selectivity and resistance to interference. The sensitivities of the sensor with rGO-PP/NF electrode for DA and 5-HT were 99.3 and 86 µA/µMcm^2^, respectively.

In the Supplementary Table S1, these results were compared with the results of some previous works. Compared with reported sensors, the performance of this work sensor showed good distinguishability in its simultaneous response to the DA and 5-HT. The improved performance might have been due to the highly catalytic active sites of rGO-PP/NF based on negatively charged Nafion surface, the large surface area, and the good electron-conducting support materials. However, challenging phase are still remained for the simultaneous and selective detection of multiple NTs including DA and 5-HT without interfering other interferents, such as epinephrine, norepinephrine, ascorbic acid, and others due to their similar redox potentials.

### Selectivity

The presence of interference is an important parameter to determine the selectivity of rGO-PP/NF for 5-HT detection. The coexistence of 5-HT with other interfering species may lead to mixed responses current due to their close oxidation potentials.

**Figure 5:**
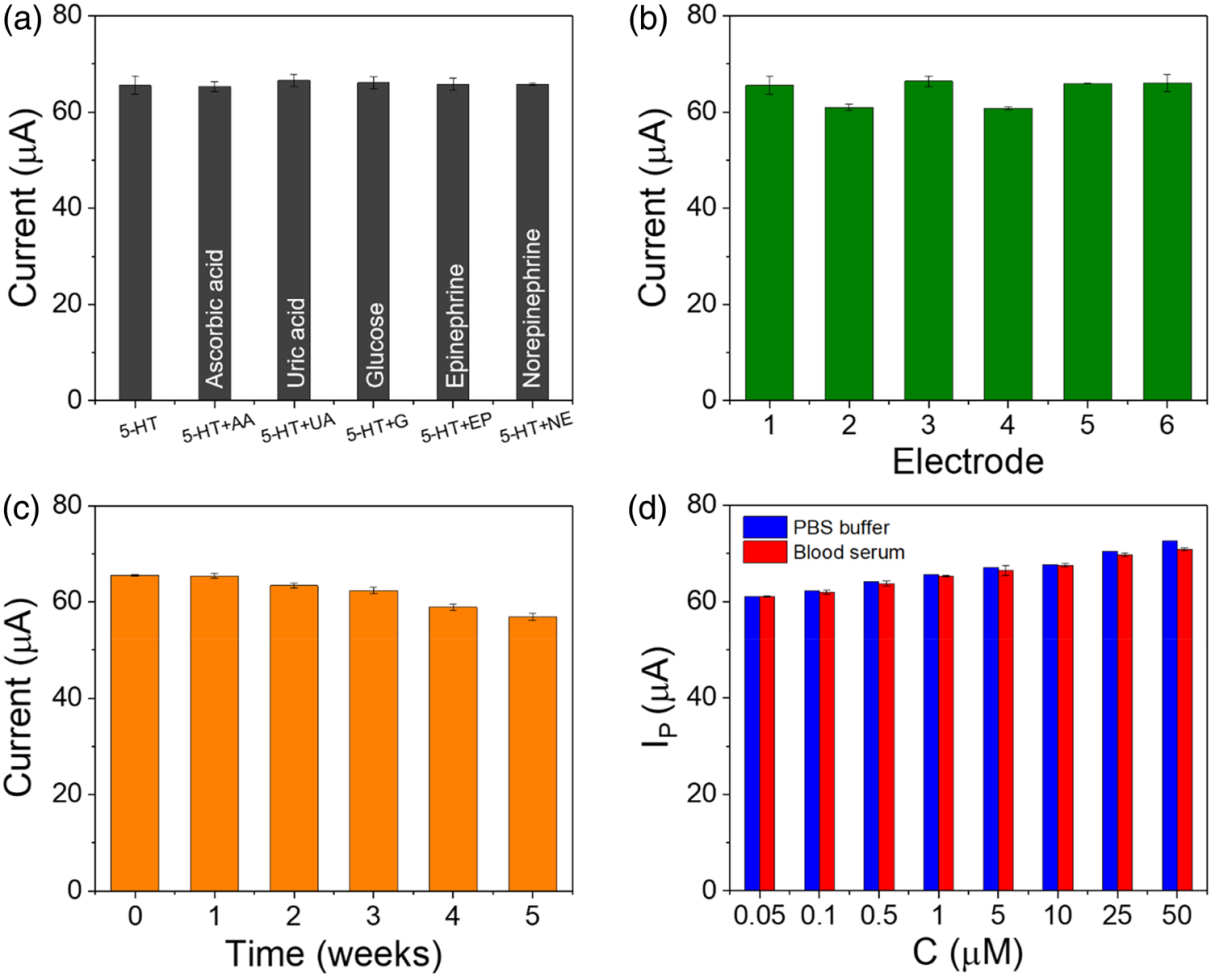
Comparison of the DPV responses of 1 μM 5-HT and 1 μM 5-HT with different concentrations of interferents (1000 μM AA, 50 μM UA, 100 μM glucose, 10 μM epinephrine, and 10 μM norepinephrine) (n = 3); (**b**) DPV responses of the fabricated six different sensors for checking reproducibility (n = 3); (**c**) DPV responses during 5 weeks for checking long-term stability; (**d**) comparison of DPV responses of the rGO-PEDOT:PSS/Nafion electrode to the different concentrations of spiked 5-HT in PBS buffer and blood serum (n = 3).

Therefore, the selectivity of the sensor with the rGO-PP/NF electrode was investigated by the DPV oxidation current response of 5-HT (1 μM). We tested ascorbic acid (AA, 1000 μM), uric acid (UA, 50 μM), glucose (100 μM), epinephrine (EP, 10 μM), and norepinephrine (NE, 10 μM) in 0.1 M PBS (pH 7.4), and the results are presented in Fig. 5a and Supplementary Table S2. In the presence of other interfering substances, the response currents small enough to be ignored compared to that of 1 μM 5-HT. Supplementary Table S2 shows the oxidation current and potential (EP) of 5-HT. The peak oxidation current of 5-HT remained 65 μA, and it showed that a higher concentration of interfering species did not interfere significantly with the response of the 5-HT DPV. Especially, at 1000 μM of AA, the oxidation potential of 5-HT has not been shifted at 0.25 V due to their repulsion from the negatively-charged Nafion surface. Based on these results, the rGO-PP/NF had higher anti-interference capability for AA, UA, glucose, EP, and NE in the 5-HT determination. This confirms the excellent selectivity of the rGO-PP/NF electrode for 5-HT.

### Reproducibility and stability of the sensor with rGO-PP/NF

The reproducibility of rGO-PP/NF was investigated by fabricating of six electrodes, and, as shown in Fig. 5b, the 5-HT oxidation peak current was measured through DPV in 0.1 M PBS (pH 7.4). Noticeably, there was a small variation with a standard deviation of only 7.84%. The small deviation indicated that the sensor was highly reproducible and suitable for mass production applications. The long-term stability also was studied for the rGO-PP/NF over the time interval of five weeks (Fig. 5c). After 5 weeks, the peak currents of 5-HT were changed from 65 to 56.6 μA. The deviation of the 5-HT oxidation peak current was 13%. These results confirmed that the rGO-PP/NF shows excellent reproducibility, repeatability, and stability as a 5-HT sensor.

### Analysis of real samples

In order to demonstrate that the sensor could be used in practical application, the sensor with the rGO-PP/NF electrode was tested, and its recovery values were compared by using real serum samples that had been spiked. As shown in Fig. 5d, the concentrations of 5-HT in the spiked human serum samples ranged from 0.05 to 50 μM (red bar) with a blank buffer (blue bar). Based on the 5-HT detection of the serum, recoveries ranged from 93.3 to 101.8% (RSD, n = 3) (Supplementary Table S3), while the sensor with rGO-PP/NF effectively was able to detect 5-HT in the sample of human serum. Interestingly, the results obtained for 0.05–5 μΜ showed a good range of recovery, which indicated that the sensor could efficiently detect low levels of 5-HT in human serum, and its performance was highly reproducible and feasible. In addition, the sensor with rGO-PP/NF was examined for the determination of 5-HT in DA. For this purpose, the concentration of 5-HT was used to detect the clinical range in serum samples. DA and 5-HT were added to the serum sample (0.1–5 μM), and the details are provided in Supplementary Table S4. The recoveries of DA and 5-HT were 89.4–93.3% and 89.1–96.1%, respectively. The RSDs of the experiment that was repeated three times were less than 5%. Hence, the rGO-PP/NF was obtained in a good range of recovery in undiluted serum samples, indicating it suitability for detecting DA and 5-HT detecting in the analysis of real samples.

## Conclusion

The hybrid interface based on the electrochemically reduced graphene oxide-PEDOT:PSS/Nafion (rGO-PP/NF) was provided a favorable interface for the simultaneous detection of dopamine (DA) and serotonin (5-HT). Placing the rGO-PP/NF onto an Au seed layer of the flexible substrate was simple, and it included the sequential electrophoretic deposition of GO, reduction at optimal pH buffer media, electropolymerization of EDOT:PSS, and Nafion coating. The optimally formed rGO at the pH 4 condition gave rise to enhanced interfacial conductivity and the decorated PEPDT:PSS maximized the electro-catalytic properties for the highly sensitive DA and 5-HT determination (DA and 5-HT sensitivities: 99.3 and 86 µA/µM cm^2^, respectively). In addition, although the negatively charged rGO-PEDOT:PSS interface is good enough for selective and simultaneous DA and 5-HT determination, the Nafion coating brought further enhancement of the negatively charged electrode interface, and it provided highly selective DA and 5-HT detection in co-existence with AA, UA, glucose, epinephrine, and norepinephrine, and it also good stability for 5 weeks. Since the similar oxidation potential among AA, DA, 5-HT, and UA that means signal-overlapping of these chemicals, therefore, the selective detection for target chemicals (DA and 5-HT) has been a main research issue. The GO/PEDOT:PSS in our previous work was not appropriated for simultaneous and selective determination of DA and 5-HT due to overlap of the oxidation peaks between DA and 5-HT. The most remarkable result was that the constructed sensor with rGO-PP/NF electrode was possible for relatively accurate and simultaneous detection of DA and 5-HT in human serum with various DA and 5-HT concentration ranges.

Finely controlled electrochemical deposition method (e.g. lowered current) of GO and electrochemical reduction process at optimal condition (e.g. optimized pH buffer and cyclic voltammetry condition at room temperature) without using any harsh thermal treatment and toxic chemical treatment is not only valuable approach but also inevitable process without damages (e.g. delamination of thin pre-patterned metal layer) for flexible sensor application with very thin patterned seed layer.

These results imply that the fabricated sensor with rGO-PP/NF electrode is promising for using multi-neurotransmitter monitoring neural electrodes or early diagnosis biochip application for simultaneous detection of multiple neurotransmitters.

## Methods

Chemicals and reagents. For fabricating the flexible sensor configuration, Polyimide (PI, VTEC 1388) was acquired from Richard Blaine International, Inc., Philadelphia, PA, USA. DNR-L300-30 was obtained from Dongjin, Seoul, Korea. AZ 9260 was acquired from AZ Electronic Materials, NJ, USA. Phosphate buffer saline (0.1 M PBS, pH 7.4) was obtained from Duksan General Science in Korea. For electrochemically reduction, a phosphate buffer solution that contained K2HPO4 and KH2PO4 was adjusted to the desired pH. DA, 5-HT, ascorbic acid (AA), uric acid (UA), glucose, epinephrine (EP), and norepinephrine (NE) were purchased from Sigma-Aldrich for electrochemical analysis. Commercially-sterile, filtered human serum (from human male AB plasma, USA origin, code H4522) was also obtained from Sigma-Aldrich for the spiking test.

Fabrication of the flexible sensor. The flexible sensor includes the Au working, the counter, the reference electrode. The fabrication sequence was described in detail in our previous work^5^. The first polyimide (PI, thickness of 20 µm) was spin-coated as a substrate layer. After curing, a negative photoresist was spin-coated on top of the PI layer for the lift-off process. After patterning, Cr/Au (10/100 nm) were deposited using an e-beam evaporator. After the lift-off process, the second PI was spin-coated and cured for the insulation layer (with a thickness of 3 μm). The positive photoresist was coated on the second PI layer to open the electrode sites and the connector pads. After patterning, the exposed PI patterns were etched by reactive ion etching. A laser dicing machine was used to cut the perimeter of the defined sensor. Then, it was easy to detach the flexible sensor from the wafer.

**Figure 6:**
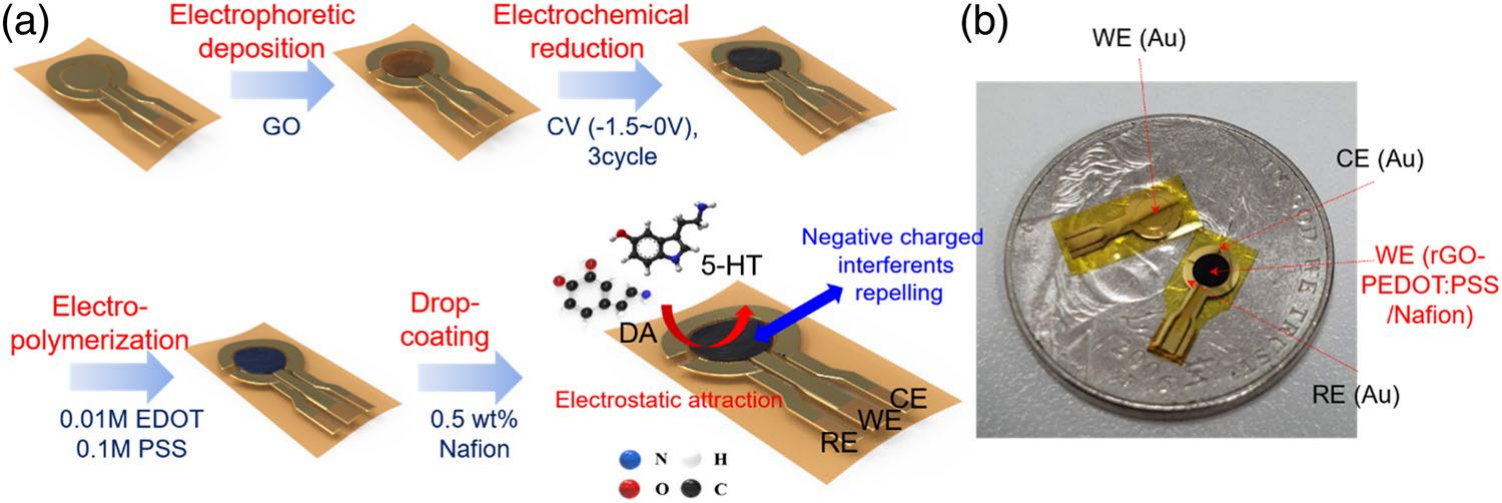
(**a**) Schematic drawing for the fabrication sequence of the sensor with rGO-PEDOT:PSS/Nafion working electrode; (**b**) photographic image of the sensors.

Preparation of the rGO-PEDOT:PSS/Nafion on the flexible sensor. Figure 6 shows the conceptual drawings for the process of fabricating the flexible sensor with the rGO-PP/NF and the photograph image of the fabricated sensors. As shown in Fig. 6, the rGO-PP/NF on a thin Au working electrode of the fabricated flexible sensor was prepared as follow. First, the GO was deposited electrophoretically with 1 μA during 1200 s. Then, the working electrode, which consisted of selectively deposited GO on thin Au, was dried for 5 h at room temperature. Second, in order to conduct the electrochemical reduction of the deposited GO, we used the deposited GO on Au working electrode, the Pt wire as a counter, and the Ag/AgCl electrodes as the reference electrodes. The electrochemical reduction was conducted in buffer solutions that had various pH values, i.e., 1.68, 4, 7.4, and 12 by cyclic voltammetry in order to determine the optimal rGO condition. The potential window was applied from − 1.5 to 0 V, and the scan rate was 50 mV/s (3 cycles). After electrochemical reduction, the color of the GO-coated electrode changed from brown to black, and the rGO was rinsed with deionized (DI) water three times and dried at room temperature. Third, the rGO was decorated with the prepared EDOT:PSS solution (0.01 M EDOT and 0.1 M PSS in deionized water) by electro-polymerization (16 μA for 300 s). Finally, the rGO/PP was coated with 0.5 wt% Nafion (1 μl).

Electrochemical characterization. The electrochemical performances of the sensors were evaluated by an Autolab (PGSTAT 302N, NOVA software, Ecochemie, Utrecht, The Netherlands) at room temperature. Three electrode configurations were used for CV, EIS, and DPV with an Au reference, Au counter, Au (2.4 mm in diameter) working electrode, and a fabricated GO-based working electrode (GO, rGO, rGO-PP, and rGO-PP/NF). The design and fabrication sequences for the sensor were described in detail in our previous work^5^

The CV with potential limits of − 0.2 and 0.8 V was performed with a scan rate of 100 mV/s, and the frequency range of EIS was from 1 to 105 Hz. The parameters of the DPV measurements were set as follows, i.e., the scan rate was 25 mV/s, the pulse width was 0.06 s, and the amplitude was 30 mV. Fresh solutions were prepared daily, and they were kept in the dark at the temperature of 4 ℃ to avoid the natural oxidation of DA and 5-HT. All of the experiments were conducted at ambient temperature. The selectivity of the sensor with rGO-PP/NF electrode was investigated by DPV oxidation current response of 5-HT (1 μM). We tested ascorbic acid (AA, 1000 μM), uric acid (UA, 50 μM), glucose (100 μM), epinephrine (EP, 10 μM), and norepinephrine (NE, 10 μM) in 0.1 M PBS (pH 7.4). The reproducibility of rGO-PP/NF was investigated by six electrodes and 5-HT oxidation peak currents. In order to evaluate the reproducibility and selectivity in the detection of 5-HT in 0.1 M PBS (pH 7.4), DPV was taken with 1 μM of 5-HT and the calculated (n = 3).

Surface morphologies and elemental analyses. The surface morphologies and elemental analyses of the electrode were evaluated by scanning electron microscopy (SEM, Regulus8230). The Fourier-transform infrared (FT-IR) spectra were captured using a Thermo Nicolet iS10 spectrometer, and the pellets were made by KBr for FT-IR analysis. The Raman spectra were recorded using a Renishaw Raman Microscope with a neodymium-doped yttrium aluminum garnet laser with a 532 nm wavelength photon beam. X-ray photoelectron spectroscopy (XPS, Ulvac, Japan) was investigated by a monochromatic Al Kα X-ray source. The XPS depth profiling was performed to obtain the approximate thicknesses of the layers. A high-energy Ar^+^ ion beam with accelerated voltage of 2 kV was used to sputter the samples layer by layer from the top surface within an area of 1 × 1 mm^2^. After each consecutive sputter cycle, the regions for the Au4f, C1s, F1s, O1s, and S2p peaks were registered and analyzed.

Serum spiking test. In order to investigate of the practical validation of the sensor with rGO-PP/NF for the simultaneous detection of DA and 5-HT, all of the serum samples were prepared without any other further treatment or dilution. DA and 5-HT of various concentrations were tested in spiked serum samples. In order to obtain the quantitative analysis, the DPV current responses of the sensor with the rGO-PP/NF electrode were checked and compared at a standard 5-HT solution and 5-HT spiked serum by DPV. The measured current responses from the standard solution and the spiked serum with various DA and 5-HT concentrations were calculated and compared by using the percent recovery (% recovery = C_i_/C_o_/C_x_), where, C_i_ and C_o_ are the experimentally obtained concentrations of 5-HT in the spiked and blank serum samples, respectively, and C_x_ is the concentration of the 5-HT that actually was spiked into the serum samples. The study was approved by the institutional review boards of Seoul National University Hospital (SNUH IRB No. H-2103-117-1206). All experiments were performed in accordance with relevant guidelines and regulations.

### Supplementary Information

Supplementary Information.

## Data Availability

The datasets used and/or analysed during the current study available from the corresponding author on reasonable request.
