# Supplementary material for: Electrodeposited reduced graphene oxide-PEDOT:PSS/Nafion hybrid interface for the simultaneous determination of dopamine and serotonin
Source: Sci Rep. 2023 Nov 20;13:20274. doi: 10.1038/s41598-023-47693-6 (PMC10662300; doi:10.1038/s41598-023-47693-6)

**Electrodeposited Reduced Graphene Oxide-PEDOT:PSS/Nafion Hybrid Interface for the Simultaneous Determination of Dopamine and Serotonin**

Seung Hyeon Ko^1, 2^, Seung Wook Kim^2^, Soo Hyun Lee^1^, and Yi Jae Lee^1,3*^

^1^Brain Science Institute, Korea Institute of Science and Technology, Seoul, 02792, South Korea

^2^Department of Chemical and Biological Engineering, Korea University, Seoul, 02841, South Korea

^3^Division of Bio-Medical Science & Technology, KIST School, University of Science & Technology (UST), Seoul, 02792, South Korea

**(Supporting information)**

*Corresponding author email: [yijaelee@kist.re.kr](mailto:yijaelee@kist.re.kr)

**Supplementary Fig. S1.** XPS spectra of electrochemically deposited rGO-PP electrode on Au. (a-d) GO, (e-h) rGO, (i-l) rGO-PP. Fitted peaks for C1s around 280-292 eV (in a red square), O1s (green) peaks from 526 to 538 eV, and S2p (blue) peaks from 162 to 174 eV, respectively.

**
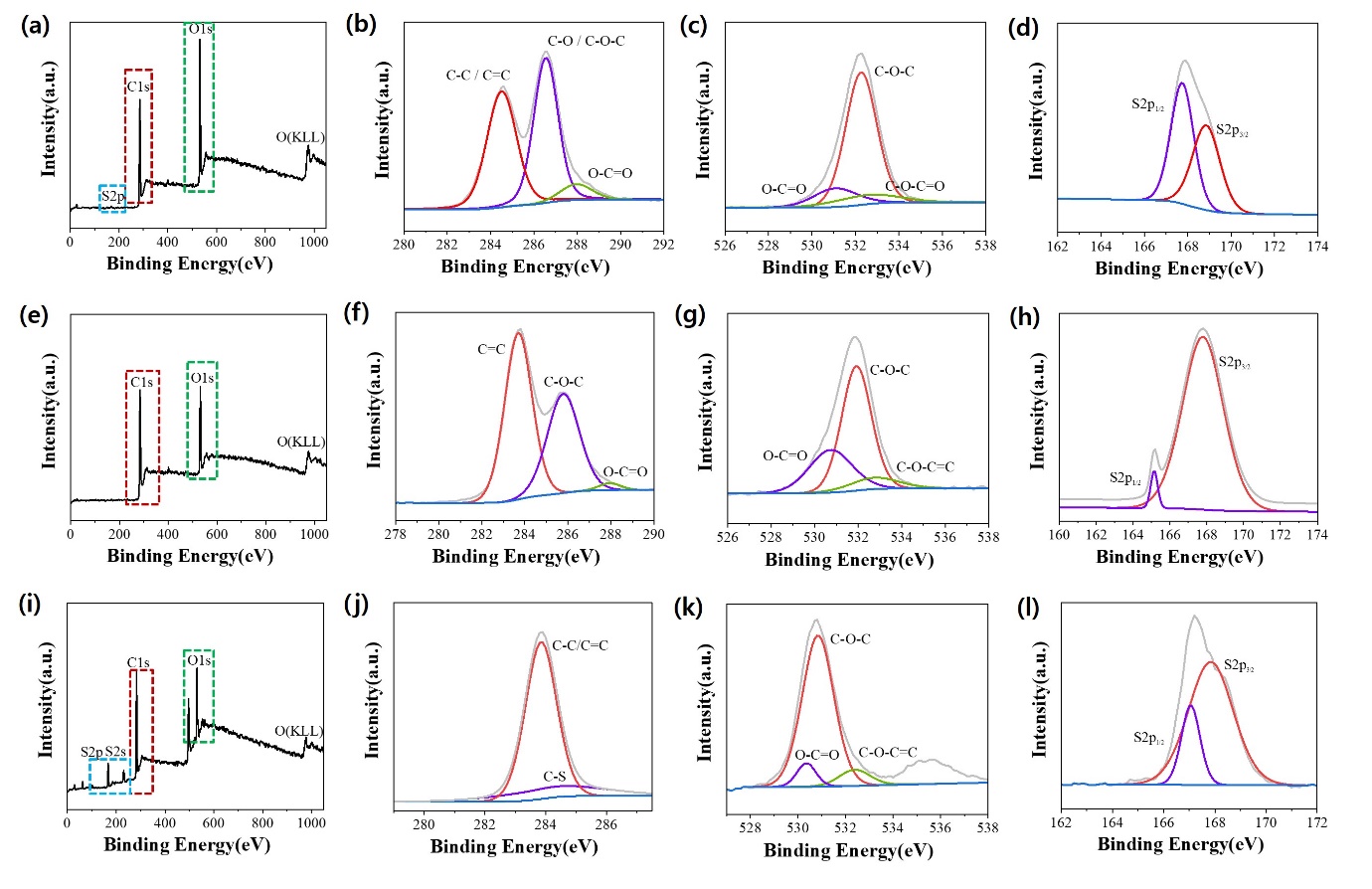
**

We checked the XPS spectrum according to the rGO-PP electrode electro-plating step. The GO and rGO show general XPS data in which C=C sp2 bond peak intensity increases and C-O and O-C=O both peak intensity decreases in C1s. And, what we should focus on is the XPS analysis result of rGO-PP. The most prominent part of C1s of rGO-PP is the C-S peak, which is the result of the C-S bond appearing due to the influence of S present in the PP molecule. In addition, it can be seen that the intensity of S2p increases. As shown in fig. a and e, it can be seen that the intensity of S2p is weak, but as shown in fig. i, the peak of S2p is stronger than that of GO and rGO. Looking at the results of fig. i-l, it can be confirmed that the electro-plating of rGO-PP electrodes we produced was successful.

**Supplementary Fig. S2.** Cross-sectional SEM image of the rGO-PP/NF. All images display an 10,000× magnification of the electrode surfaces. The scale bars represent 2 µm.

**
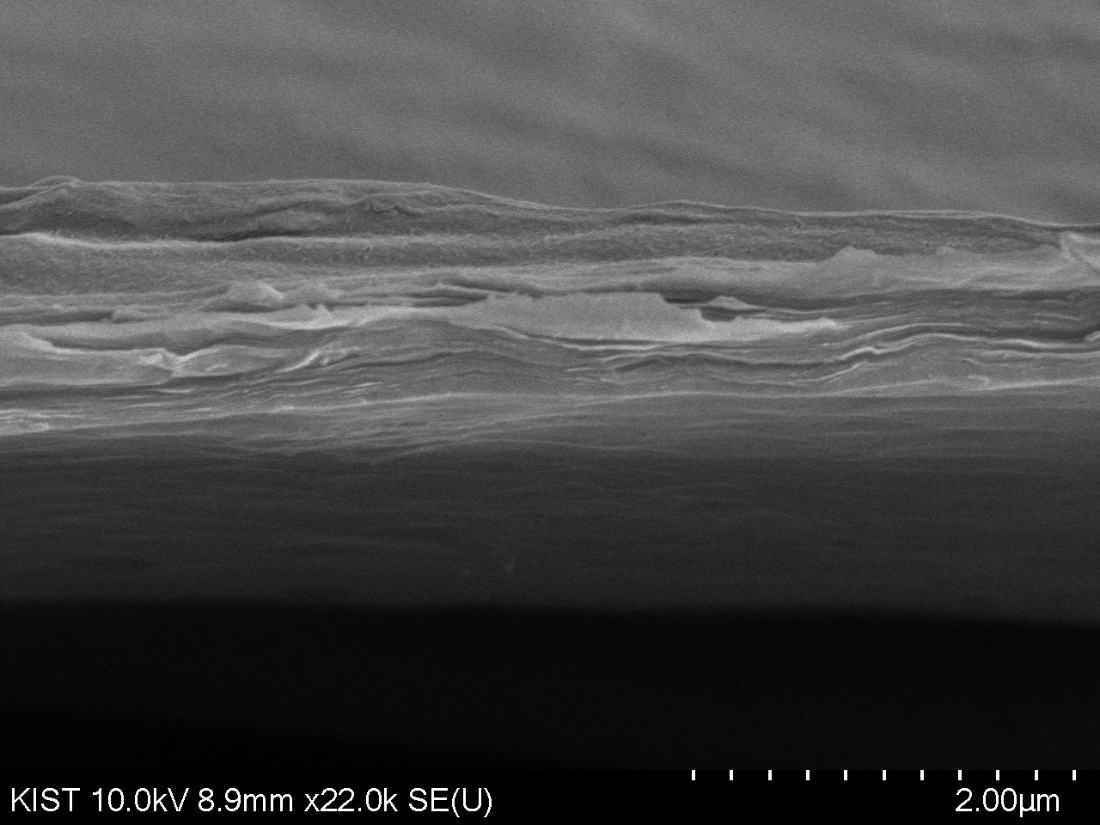
**

**Supplementary Table. S1.** Comparison of the different type reduced graphene oxide or PEDOT:PSS modified electrode for electrochemical detection of 5-HT.


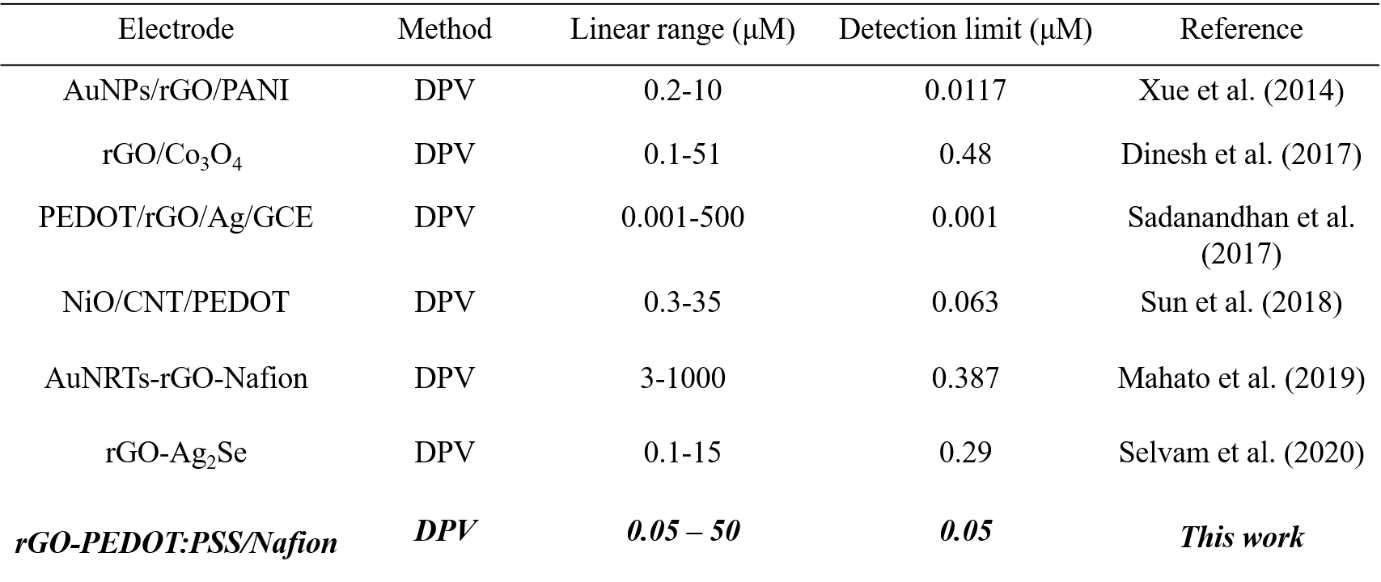


**Supplementary Table. S2.** Interference study in the electrochemical detection of serotonin.


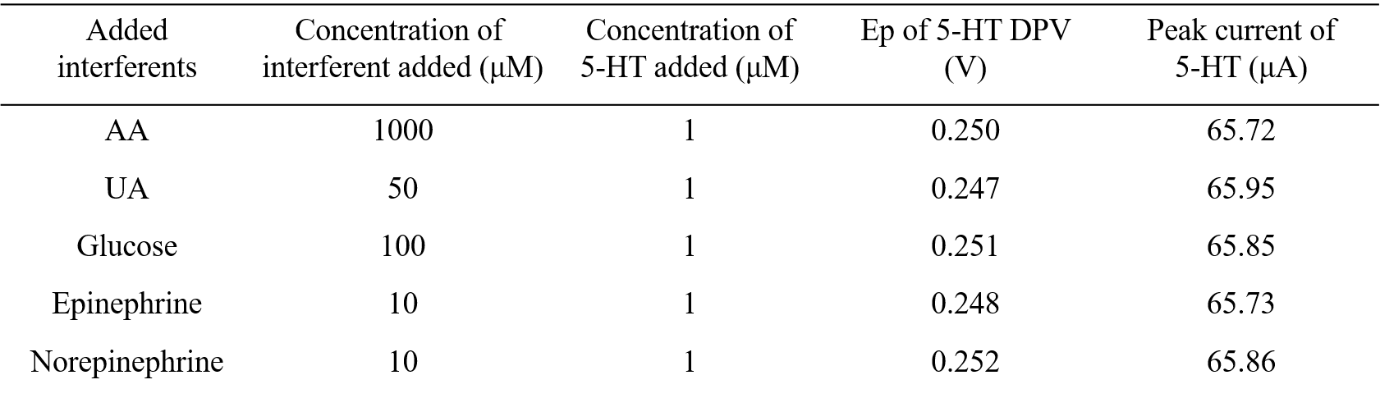


**Supplementary Table. S3.** Detection of serotonin in serum samples.


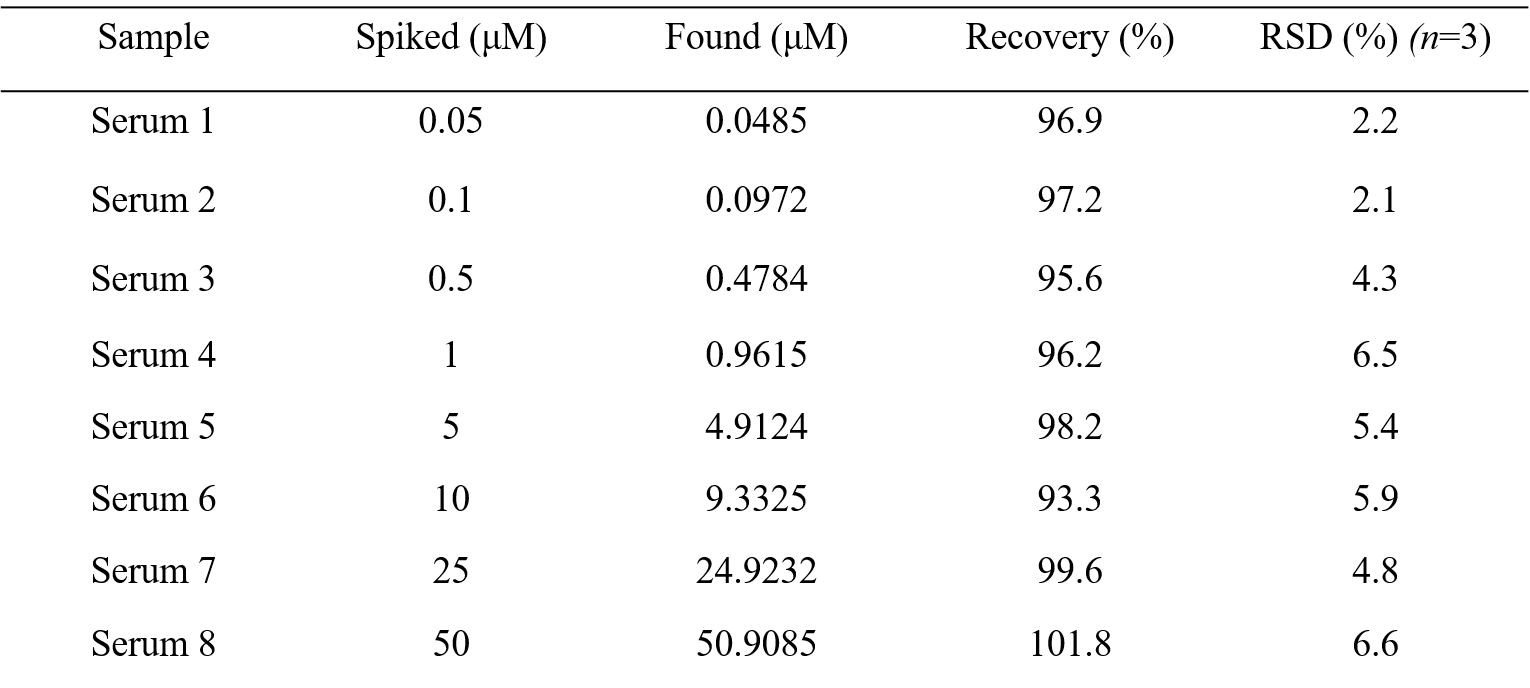


**Supplementary Table. S4.** Detection of dopamine and serotonin in serum samples.


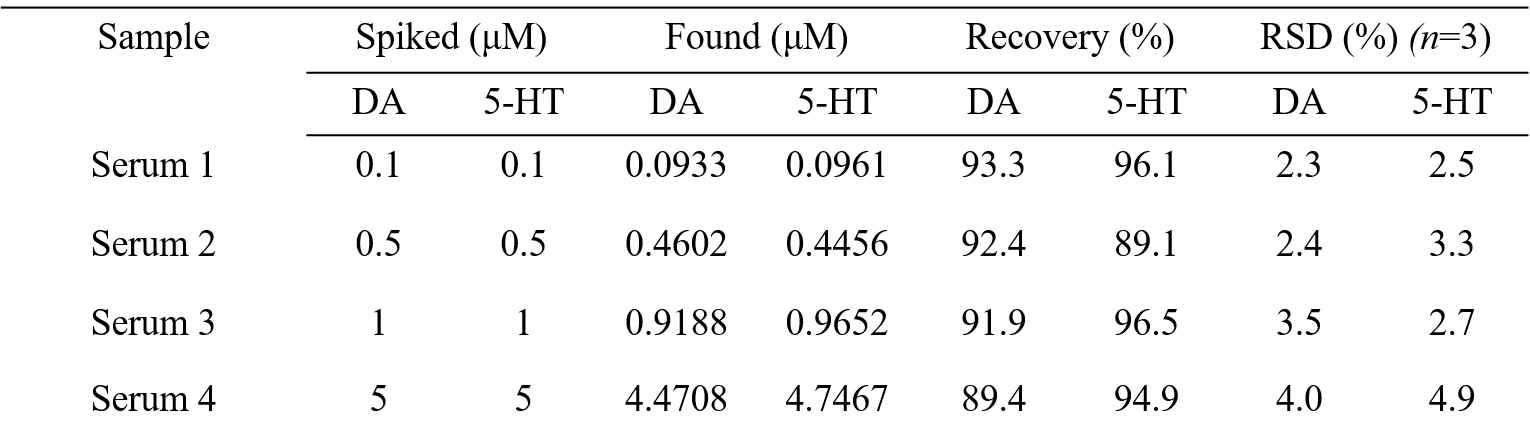

Supplement: Supplementary file 1 — Supplementary Information. [file 41598_2023_47693_MOESM1_ESM.docx]
